# Ourselves as Others See Us

**Published:** 1889-12

**Authors:** 


OURSELVES AS OTHERS SEE US.—THE BUFFALO MEDICAL AND SURGICAL JOURNAL has just begun its twenty-ninth volume, and has combined with or absorbed the *Medical Press of Western New York*, published in this city. The BUFFALO MEDICAL AND SURGICAL JOURNAL has been enlarged, and, since its present editors assumed charge of its affairs, improved in every way, and is now one of the leading medical journals in the country.—*Dental Advertiser*, (Buffalo,) Oct., 1889.

